# Climate change, forced pastoralist migration, and zoonotic spillover in Somalia: a neglected one health threat to regional health security

**DOI:** 10.1016/j.onehlt.2026.101585

**Published:** 2026-09-15

**Authors:** Ayuub Ahmed Osman, Maryan Ali Adam, Ilyas Abdullahi khalif

**Affiliations:** Faculty of Health Sciences and Tropical Medicine, Somali National University, Mogadishu, Somalia; Banadir Research, Consultancy & Evaluation Center, Mogadishu, Somalia

**Keywords:** Climate change, Pastoralist migration, Zoonotic diseases, One health, Regional health security

## Abstract

Climate change is reshaping pastoralist mobility across Somalia, creating overlooked pathways for zoonotic disease emergence. Recurrent droughts, floods, and environmental degradation force livestock-dependent communities into new ecological interfaces where humans, livestock, wildlife, and vectors increasingly interact. Weak surveillance, fragile health systems, and limited cross-border coordination further heighten outbreak risks. Strengthening One Health surveillance through community-based reporting, mobile health and veterinary services, integrated laboratory systems, and regional data sharing is essential to improve early detection, reduce spillover risk, and strengthen health security in the Horn of Africa.

Climate-driven pastoralist migration appears to be a neglected pathway for zoonotic spillover in Somalia, and the supplied literature supports framing it as a regional health security blind spot rather than only an animal health problem [1], [2].

Somalia's economy and livelihoods remain deeply tied to livestock, with the sector employing about 65% of the population, generating 40% of GDP, and supplying more than 80% of hard currency [1]. Pastoralists depend heavily on livestock and commonly practice transhumance, making human–animal contact structurally intimate and climate-sensitive [1]. Somalia has experienced repeated climate shocks, including recurrent droughts and floods, and climate variability is associated with migration pressure in the country [2]. Across the wider Horn, drought has caused extreme livestock loss and large migration out of affected border areas, including the Ethiopia–Kenya–Somalia frontier.

Somalia's climate crisis is therefore not only shrinking pasture and water; it is reorganizing where people and animals move, who they mix with, and which health systems they can still access. That mobility pathway is the missing link between climate adaptation and outbreak prevention.

Repeated droughts, floods, pasture degradation, and water scarcity force herders to move livestock to better grazing areas or relocate altogether [2]. Environmental disruption and cross-border movement intensify contact among humans, livestock, wildlife, and vectors, which increases opportunities for spillover and spread. In pastoral settings, mobility does not produce a single uniform effect: it can mitigate some risks, but forced immobility or blocked grazing routes can also increase exposure by preventing avoidance of seasonal vectors such as mosquitoes and tsetse flies [3]. The strongest formulation is therefore not that “pastoralists spread disease,” but that climate-disrupted mobility systems reshape exposure ecologies in ways that can favor zoonotic transmission.

That mechanism is plausible for several pathogens already documented as relevant to Somalia and pastoral Africa. Somalia's zoonoses literature identifies 22 reported zoonotic infections, with Rift Valley fever, brucellosis, and hepatitis E most frequently studied [1]. Close livestock contact raises zoonotic risk in Somali pastoralists [1]. Regional evidence also identifies brucellosis, Q fever, and Rift Valley fever as major human–livestock–wildlife pathogens, and pastoral meta-analysis shows Rift Valley fever and Crimean-Congo hemorrhagic fever are endemic across pastoral systems, with highest prevalence often at livestock farms and value-chain nodes [4]. Anthrax and Rift Valley fever are also repeatedly recognized as important zoonoses in East African livestock systems and One Health prioritization exercises.

The policy blind spot is surveillance. In Somalia, accessible health services are limited by pastoral remoteness, mobility, and a fragile health system [1]. Existing zoonoses evidence is thin, largely descriptive, often derived from non-resident populations, and supported by very limited in-country laboratory analysis [1]. More broadly, surveillance in low-income settings still prioritizes urban or adjacent rural populations, leaving remote rural communities with frequent animal contact comparatively unsupported despite their importance for global health security [5]. Conventional systems are also siloed: human and animal surveillance often remain weakly integrated, laboratories are disconnected from epidemiologic systems, and core detection and data-management functions are still performed separately or with low outbreak sensitivity [6]. Where mobility is cross-border, delayed or incomplete data sharing further undermines early warning for mobile populations.

A credible response is available in the same literature. One Health surveillance should extend beyond pathogen detection to include the drivers of emergence, including environmental and mobility signals [6]. Community-based syndromic surveillance in pastoral settings is feasible and can generate relevant human and animal health data in remote areas with poor service access [7]. For example, community-based symptom reporting among agro-pastoralists in Chad demonstrated that trained community members can contribute linked human and livestock health information in remote settings, providing an early warning mechanism where conventional surveillance systems are limited [7]. Reviews focused on remote rural surveillance recommend training frontline workers and clinicians in One Health practice, creating zoonotic disease units, and equipping remote services to collect linked human, animal, and environmental data [5]. In In Somalia, such approaches could be adapted through mobile health and veterinary teams operating along pastoral migration routes, integrating livestock health assessments, human disease reporting, vaccination activities, and environmental monitoring to improve detection among populations that are frequently missed by fixed health facilities. Additional priorities include mobile veterinary and health services, community reporting, cross-border data-sharing mechanisms, stronger laboratory capacity, and surveillance along livestock movement and value-chain nodes [1]. At the regional level, coordinated surveillance across shared pastoral corridors, including the Ethiopia–Kenya–Somalia frontier, could improve early detection of climate-sensitive zoonoses and facilitate timely information exchange between countries [2], [6].

Climate-driven pastoralist migration has therefore become an under-recognized determinant of zoonotic spillover in Somalia. If regional health security strategies continue to treat mobile pastoralists as peripheral to surveillance design, they will keep missing one of the clearest climate-sensitive interfaces where the next outbreak can emerge.

## CRediT authorship contribution statement

**Ayuub Ahmed Osman:** Supervision. **Maryan Ali Adam:** Writing – review & editing. **Ilyas Abdullahi khalif:** Writing – review & editing.

## Ethical approval

This article did not involve human participants, human data, or animals; therefore, ethical approval was not required.

## Funding

The authors received no specific funding for this work.

## Declaration of competing interest

The authors declare that they have no known competing financial interests or personal relationships that could have appeared to influence the work reported in this paper.

## Data Availability

No datasets were generated or analyzed during the current study.
